# Institutionalizing health technology assessment in Ethiopia: seizing the window of opportunity

**DOI:** 10.1017/S0266462323000454

**Published:** 2023-07-21

**Authors:** Daniel Erku, Damian Walker, Ana A. Caruso, Befikadu Wubishet, Yibeltal Assefa, Samuel Abera, Alemayehu Hailu, Paul Scuffham

**Affiliations:** 1 Centre for Applied Health Economics, School of Medicine, Griffith University, Brisbane, QLD, Australia; 2 Menzies Health Institute Queensland, Griffith University, Brisbane, QLD, Australia; 3 Centre for Research and Engagement in Assessment of Health Technology (CREATE), Addis Ababa, Ethiopia; 4 Health Financing, Technologies, and Market Dynamics, Global Health Systems Innovation, Management Sciences for Health, Arlington, VA, USA; 5 Centre for Economic Impacts of Genomic Medicine, Macquarie University, Sydney, NSW, Australia; 6 School of Public Health, The University of Queensland, Brisbane, QLD, Australia; 7 Healthcare Financing Technical Advisor Partnership and Coordination, Directorate, Ministry of Health, Addis Ababa, Ethiopia; 8 Department of Global Public Health and Primary Care, Bergen Center for Ethics and Priority Setting, University of Bergen, Bergen, Norway; 9 Harvard T.H. Chan School of Public Health, Harvard University, Boston, MA, USA

**Keywords:** health technology assessment, sub-Saharan Africa, decision making, capacity building, policy making, priority setting

## Abstract

Ethiopia’s commitment to achieving universal health coverage (UHC) requires an efficient and equitable health priority-setting practice. The Ministry of Health aims to institutionalize health technology assessment (HTA) to support evidence-based decision making. This commentary highlights key considerations for successful formulation, adoption, and implementation of HTA policies and practices in Ethiopia, based on a review of international evidence and published normative principles and guidelines. Stakeholder engagement, transparent policymaking, sustainable financing, workforce education, and political economy analysis and power dynamics are critical factors that need to be considered when developing a national HTA roadmap and implementation strategy. To ensure ownership and sustainability of HTA, effective stakeholder engagement and transparency are crucial. Regulatory embedding and sustainable financing ensure legitimacy and continuity of HTA production, and workforce education and training are essential for conducting and interpreting HTA. Political economy analysis helps identify opportunities and constraints for effective HTA implementation. By addressing these considerations, Ethiopia can establish a well-designed HTA system to inform evidence-based and equitable resource allocation toward achieving UHC and improving health outcomes.

## Introduction

The 2030 sustainable development goals (SDGs), adopted by the United Nations in 2025, represent a global commitment to sustainable development and aim to address pressing global challenges. One of the key targets of the SDGs is achieving universal health coverage (UHC) by 2030 (1). UHC means that everyone should have access to quality healthcare services without facing financial hardship. Achieving UHC requires significant progress toward efficient, accessible, quality, and equitable health services while ensuring financial risk protection for households burdened with significant healthcare expenses (2). However, many low- and middle-income countries (LMICs) face significant budgeting and financial constraints, making it difficult to implement their plans toward UHC (2;3). LMICs often have limited fiscal space for health, rely on donor funding for healthcare, and face significant out-of-pocket (OOP) payments by households. Additionally, the allocation of limited healthcare resources can be inefficient and inequitable, further exacerbating access to quality healthcare services.

In Ethiopia, the healthcare sector is funded by various sources including international loans and donations (35 percent), the Ethiopian Government (32 percent), and OOP payments (31 percent) (4). Despite an increase in health financing from domestic sources from US$1.3 billion in 2008 to US$3.1 billion in 2017, and an increase in per capita health expenditure from US$4.50 in 1995 to US$33.2 in 2017, the amount is still inadequate compared to the recommended spending for essential health services (5;6). Furthermore, Ethiopia’s health spending constituted 5.6 percent of the gross domestic product (GDP) in the last decade, which is below World Health Organization (WHO)’s average estimation of 7 percent for low-income countries (7).

OOP payments in Ethiopia account for more than 30 percent of all health spending, which is above the average for sub-Saharan Africa according to the WHO’s Global Health Expenditure database (8). This heavy reliance on OOP payments can discourage individuals with low socioeconomic status from seeking and accessing healthcare services (9). Donor funding is another major source of funding in Ethiopia, often channeled toward priority programs such as immunization, infectious diseases, and maternal and child health. However, such funds often depend on donor preference and priorities, making it challenging to predict the level, flow, and use of resources. Although most health programs prioritized by donors are fundamental to achieving UHC, they are often delivered through vertical structures and can raise efficiency and sustainability issues, particularly in the absence of additional funding.

Efforts toward achieving UHC and the broader SDGs require a systematic approach to allocating scarce healthcare resources (10). This involves determining the financial requirements for UHC, deciding on a feasible benefits package given resource constraints, and identifying priorities for expanding service coverage. Institutionalizing health technology assessment (HTA) is critical to addressing these challenges. HTA is a process of evaluating the clinical effectiveness, safety, cost-effectiveness, ethical, and social implications of healthcare interventions (10). It provides evidence-based information to inform healthcare decision making, such as whether a particular intervention should be adopted, reimbursed, or covered by health insurance. HTA helps set priorities for health interventions, estimates the impact on budgets, and identifies potential barriers to uptake that might exacerbate inequities. The WHO has recognized the importance of HTA and declared resolutions to adopt it as an essential approach to address global health challenges, improve health systems, and achieve SDGs (11). Resolution WHA67.23 calls for the institutionalization of HTA within national frameworks to promote evidence-based policy development and decision making in health systems (11).

HTA has been widely adopted as a means of priority setting in high-income countries such as the UK, Australia, and Canada, as well as in upper-middle-income countries such as Thailand, Brazil, and Mexico (12). In recent years, there has been growing interest in HTA among sub-Saharan African countries, including Ethiopia, driven in part by commitments to UHC and the changing landscape of donor funding. However, despite this interest, HTA awareness and capacity remains low, and the use of HTA in policy making is often uncoordinated and disconnected (13). In this commentary, we argue that the institutionalization of HTA in Ethiopia is influenced not only by a lack of awareness and capacity, but also by the broader context of public-sector decision making. To understand the policy and political dynamics that affect the adoption and implementation of HTA, we applied John Kingdon’s Multiple Streams Framework (MSF), which has been successfully used to explore the political prioritization of public health issues in LMICs (14). The multiple streams in this approach are named for the parallel streams of problem, policy, and politics, which contain actors and ideas that make up the milieu from which policies rise to prominence in the agenda-setting process. The development and convergence of these streams make a policy more likely to rise onto the government’s policy agenda. Therefore, policy decisions occur at the confluence of problem, politics, and policy streams in the case study at hand, which is HTA institutionalization in Ethiopia.

## HTA and priority-setting approaches in Ethiopia

In Ethiopia, priority setting for healthcare is made at various levels of government including the national, regional, district, and service delivery levels. Over the past few decades, the country has focused on decentralizing healthcare and prioritizing health promotion, disease prevention, and basic curative services. To achieve this, the government has produced a range of documents, such as the Essential Health Service Package (EHSP) (15), to inform service delivery and reimbursement. The priority-setting approaches used during the development and revision of the EHSP were participatory, inclusive, evidence-based, and guided by a clear roadmap (16). The revision committee applied explicit sets of criteria, in the form of multi-criteria decision analysis, to systematically synthesize and collate evidence on clinical effectiveness, cost-effectiveness, equity, and budget impact of health interventions. The use of multi-criteria decision criteria in the EHSP revision and consideration of evidence from economic evaluations indicates a growing interest in and use of (economic) evidence to inform health policies in Ethiopia. Although the use of multi-criteria decision analysis and evidence-based prioritization in Ethiopia’s priority setting is a positive step forward, there are still several limitations that need to be addressed in order to enhance the effectiveness of the approach.

### Lack of relevant data and limited technical capacity

In Ethiopia, there is a lack of relevant country-specific data and the types of evidence generated do not always align with policy needs, which presents a challenge to effective priority setting. A review of published HTA reports and health economic evaluations conducted in Ethiopia identified no comprehensive HTA reports and only thirty-four full health economic evaluations published until May 2021 (17). Although some of these economic evaluations were used as inputs in the 2019 revision of the EHSP, the lack of context-relevant data to conduct contextualized cost-effectiveness analyses resulted in many of the input parameters being derived from systematic reviews (16;17). The reliance on such evidence can be problematic, as it may not accurately reflect the local health system, demographics, or epidemiology, limiting the transferability of cost-effectiveness ratios. It is therefore crucial to generate, collate, and archive country-specific data in Ethiopia to produce actionable and readily interpretable evidence for policymakers. This will not only facilitate more effective priority setting but also reduce the dependence on economic evaluations conducted in different health systems.

Another significant factor that hinders effective priority setting is the lack of local technical capacity in Ethiopia to undertake and interpret economic evaluations across the health sector. HTA development process often requires a combination of a broad mix of skills, including expertise in medicine, epidemiology, biostatistics, economics, psychology, and health outcomes research fields. Unfortunately, many HTA-related capacity-building activities in Ethiopia focus solely on technical assistance and do not adequately consider individual, organizational, and institutional aspects of capacity-building, limiting the effectiveness and sustainability of these efforts.

### Lack of funding and coordination

There is a severe lack of local research funding for conducting health economic evaluations (17). More than half of the studies included in the Erku *et al.* review of economic evaluation conducted in Ethiopia (17) were funded by non-governmental organizations or foreign institutions and focused mainly on communicable disease and maternal and child health, reiterating the need for more funding, both from the government and external funding agencies, to generate reliable, policy-relevant economic evidence in major chronic diseases. The lack of research funding also raises concerns about the sustainability of the EHSP revision exercise, which was made possible with the technical and financial support from Disease Control Priorities–Ethiopia (DCP-Ethiopia) project (16). This highlights the need for greater investment in local capacity-building initiatives to enhance the technical expertise required to generate and interpret contextualized data to inform HTA in Ethiopia, and for the government to prioritize funding for health economic research.

In addition to the challenges related to funding, data, and capacity, the lack of coordination and institutionalization has resulted in uncoordinated and fragmented efforts (18). Weak institutional arrangements and low awareness of HTA among policymakers have also been identified as critical barriers to the institutionalization of HTA and the priority-setting process in Ethiopia. Moreover, apart from the EHSP, there is little to no clear evidence on the priority-setting criteria and methods used in other key policy documents such as the Pharmaceuticals Procurement List (used by Ethiopian Pharmaceutical Supply Agency) and the Health Benefits Package (used by Ethiopia’s health insurance agency) (18). These limitations of the current priority-setting approach underscore the need to establish sustainable, locally-owned mechanisms for effective, equitable, and sustainable resource allocation in Ethiopia beyond donor funding and demand-driven technical support. Such mechanisms should be informed by a robust institutional framework and a coordinated approach to HTA and priority setting.

## Institutionalizing HTA and evidence-informed priority setting

To address the challenges in Ethiopia’s health priority-setting process, the Ministry of Health is taking steps to develop a national HTA roadmap based on evidence-based recommendations from a situation analysis. The aim is to strengthen the existing HTA-related activities in the Ethiopian health system in a more institutionalized and harmonized way as well as working toward having a strong HTA body in Ethiopia. In the following sections, we briefly discuss: (i) examination of the institutional context and power dynamics between stakeholders (political economy analysis [PEA]), (ii) human resources and capacities for HTA and (iii) a reflection on the experiences and lessons derived from healthcare systems of a comparable nature. Drawing on existing evidence (19), the integration of these aspects with a comprehensive analysis of the healthcare system and current decision making methodologies should form the foundation for an evidence-based HTA strategy. This grounding paves the way for a robust and sustainable practice of evidence-based priority setting.

### The political economy of HTA institutionalization

The process of priority setting in healthcare is complex and involves multiple stakeholders with diverse interests, preferences, and expectations. Reforms aimed at making priority setting more explicit must take into account the broader socio-economic fiscal, and political governance contexts in which stakeholders operate. These contexts can influence the governance of HTA and the priorities and feasibility of improving it. For example, when deciding on benefit package specifications, policymakers must consider heterogeneity in preferences and expectations, which involves multiple stakeholders within or across different sectors, each with different interests that may not necessarily align. To achieve effective priority setting, policymakers must navigate these complex contexts and work to balance competing interests and goals.

The formulation, adoption, and institutionalization of HTA are inherently political, and thus, policymakers should incorporate PEA and strategies into policy design, adoption, and implementation. PEA provides a lens through which to understand the structural and socioeconomic conditions underpinning decision making and conflicting interests. It combines the economics of reforms with the politics of change by examining how power and resources are distributed and how interests, incentives, and institutions enable or hinder change. PEA can be conducted by segmenting stakeholders into distinct subgroups, such as interest group politics, bureaucratic politics, budget politics, leadership politics, beneficiary politics, and external actor politics (20). By doing so, policymakers can identify which stakeholders are most likely to be engaged and consider the political feasibility of specific reforms. PEA can provide insights into how HTA and priority-setting process may evolve in response to broader health financing and health system reforms. The findings can help policymakers understand how the political, structural, and socioeconomic contexts shape the development and implementation of HTA and identify the extent to which agencies transform decision making and displace irrationality in the system. Moreover, incorporating PEA into reform processes can help policymakers develop more effective approaches to navigate the political challenges that arise when introducing policy change and ensure that priority setting is more explicit, equitable, and sustainable.

### Human resources and capacities for HTA

Sustainable improvements in the performance of key HTA-related initiatives require more than just on-demand technical assistance and knowledge transfer at the individual level. It is also important to establish robust technical and operational systems that can carry HTA work forward into the future. This includes developing local technical capacity to undertake and interpret economic evaluations, collecting relevant country-specific data, and establishing strong institutional arrangements that support HTA and priority-setting processes. Finally, it is important to learn from countries with comparable healthcare systems to Ethiopia, and to translate those findings to the Ethiopian context. This includes answering key cross-cutting questions, such as why HTA is being done, what the scope of HTA is in terms of interventions, products, or services, how and by whom HTA is conducted, and how HTA is used, and what the key knowledge dissemination strategies are. By taking these considerations into account, Ethiopia can develop a sustainable and locally owned mechanism for effective, equitable, and sustainable resource allocation, which can support the achievement of UHC.

### International experiences regarding HTA institutionalization

**Figure 1.**
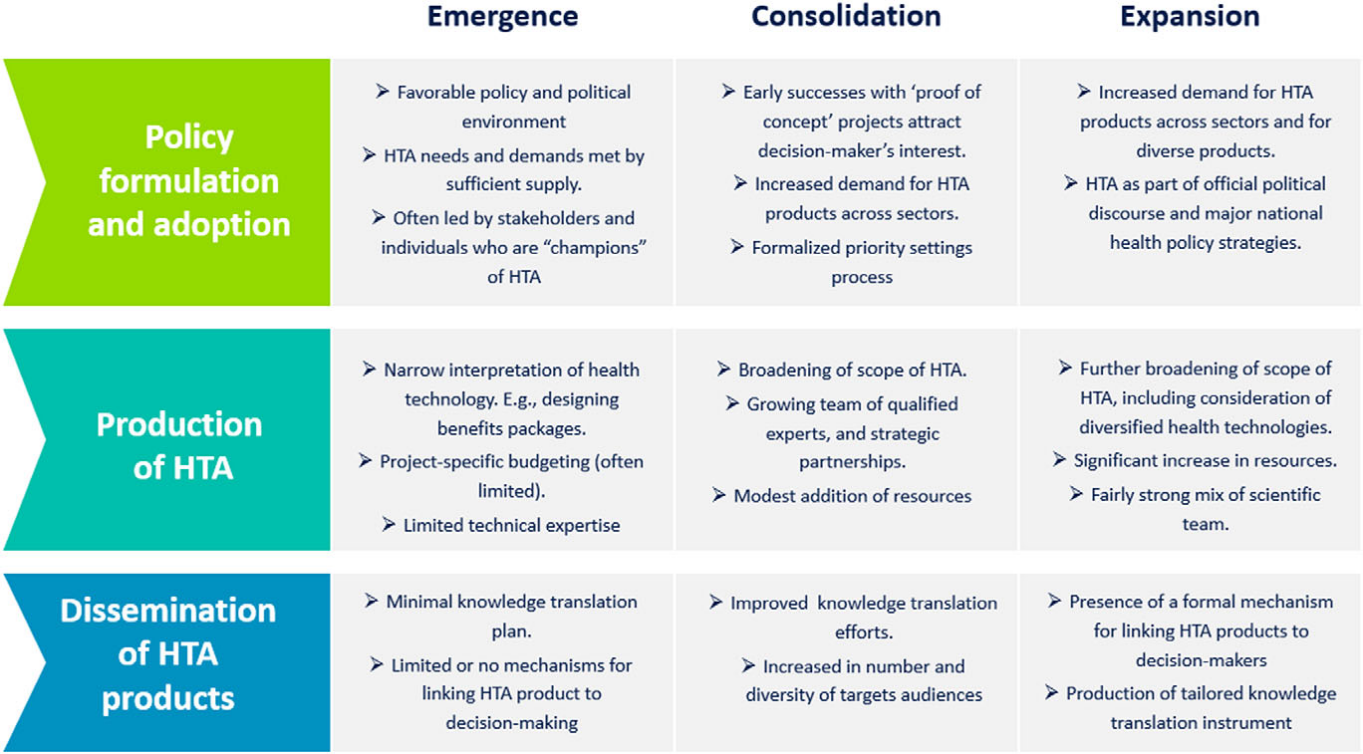
Phases of HTA development.

When developing a national HTA strategy, it is helpful to incorporate experiences and lessons from comparable healthcare systems and make deductive conclusions from international knowledge. Based on the findings from an analysis of multi-country experiences and supplemented with Battista *et al.* “natural history” of HTA (21), we provided a summary of three phases of HTA development: “emergence,” “consolidation” and “expansion” (Figure 1).

Currently, Ethiopia’s status regarding HTA development is best described as in the “emergence” phase. With Ethiopia’s recent progress toward a public insurance funding mechanism, it is crucial to employ HTA to ensure that claims and reimbursements for services and health technologies are well justified. Given that transitioning to a more structured priority-setting approach takes time, initial HTA efforts may focus on “high cost” or priority health technologies to determine the value for money and whether or not the technology should be recommended for government reimbursement or should be made widely available to the people of Ethiopia. The progression through the different phases of HTA development and the establishment of institutional and governance arrangements for HTA are interrelated with the evolution of the country’s overall health system, including the current payment mechanisms. In order to effectively transition through these phases, it is important to build on existing strengths, manage potential barriers, and create new opportunities for the meaningful and sustainable production and use of HTA products at both the policy and practice level.

International experiences from countries suggest that certain strategies can alleviate common barriers to the institutionalization of HTA. These include establishing a legal mandate for HTA, building local technical capacity through partnerships, and enhancing strategic communication with stakeholders (18). An excellent example of such initiatives is the work being done in Ghana. Before 2016, spending on medicines constituted nearly half (46 percent) of the total health expenditure in the country, 60 percent of which was being spent only on antihypertensives and antibiotics due to inappropriate prescribing, polypharmacy, and inefficient supply chain. The Ghanaian Ministry of Health, supported by an international decision support initiative, conducted an HTA study to examine the cost-effectiveness of antihypertensives (22). The findings indicated that, in the Ghanaian context, diuretics and calcium channel blockers would be more effective and less expensive than other drug classes, such as beta-blockers, angiotensin-converting enzyme inhibitors and angiotensin receptor blockers (23). This exercise saved the Ghanaian Government US$11.2 million, which is now available for hypertension prevention and early detection. Thailand is another excellent example. Efforts to use HTA methods to inform healthcare decisions in Thailand commenced by academic “champions” to manage the inefficient and inequitable distribution of limited healthcare resources. One of the most important milestones in Thailand’s HTA institutionalization journey was the establishment of Health Intervention and Technology Assessment Program (HITAP) in 2007, which has served the country’s Subcommittee for the development of the Benefits Package and Service Delivery as a technical focal point (24).

## Where to from here? Seizing the window of opportunity

As a country with limited resources, Ethiopia needs an efficient and equitable health priority-setting practice to achieve UHC. In recent years, there has been an increased interest in using explicit, evidence-based priority-setting criteria in health policy making in Ethiopia. The Health Economics and Financing Analysis (HEFA) unit and the Ethiopian Public Health Institute have been leading the way in the design and execution of various demand-driven HTA projects in Ethiopia. Despite efforts to introduce HTA, barriers such as the lack of local technical capacity, limited relevant country-specific data, weak institutional arrangements, and low awareness of HTA among policymakers still hinder the institutionalization of HTA and priority-setting processes. To address these challenges, the Ministry of Health of Ethiopia plans to introduce HTA as one of the main implementation strategies of the Health Sector Transformation Plan (HSTP II) and to produce a national HTA strategy followed by various knowledge products (25).

This commentary provides insights into the key considerations for successful formulation, adoption, and implementation of HTA policies and practices in Ethiopia. Stakeholder engagement, transparent policymaking, sustainable financing, workforce education, and political economy and power dynamics are critical factors that should be considered while developing a national HTA roadmap and implementation strategy. Ensuring effective stakeholder engagement and transparency is essential to ensure ownership and sustainability of HTA. Regulatory embedding and sustainable financing are important to ensure legitimacy and continuity of HTA production. Workforce education and training are essential for conducting and interpreting HTA, and PEA helps identify opportunities and constraints for effective HTA implementation. By establishing a well-designed and locally owned HTA system, Ethiopia can make informed decisions toward achieving UHC and improving health outcomes, building on successful experiences in other countries.
